# The role of beta band phase resetting in audio-visual temporal order judgment

**DOI:** 10.1007/s11571-024-10183-0

**Published:** 2025-01-15

**Authors:** Yueying Li, Yasuki Noguchi

**Affiliations:** Department of Psychology, Graduate School of Humanities, Kobe University, 1-1 Rokkodai- cho, Nada, Kobe, 657-8501 Japan

**Keywords:** Electroencephalography, Audio-visual integration, Temporal order judgments, Inter-trial coherence, Phase-locking index

## Abstract

The integration of auditory and visual stimuli is essential for effective language processing and social perception. The present study aimed to elucidate the mechanisms underlying audio-visual (A-V) integration by investigating the temporal dynamics of multisensory regions in the human brain. Specifically, we evaluated inter-trial coherence (ITC), a neural index indicative of phase resetting, through scalp electroencephalography (EEG) while participants performed a temporal-order judgment task that involved auditory (beep, A) and visual (flash, V) stimuli. The results indicated that ITC phase resetting was greater for bimodal (A + V) stimuli compared to unimodal (A or V) stimuli in the posterior temporal region, which resembled the responses of A-V multisensory neurons reported in animal studies. Furthermore, the ITC got lager as the stimulus-onset asynchrony (SOA) between beep and flash approached 0 ms. This enhancement in ITC was most clearly seen in the beta band (13–30 Hz). Overall, these findings highlight the importance of beta rhythm activity in the posterior temporal cortex for the detection of synchronous audiovisual stimuli, as assessed through temporal order judgment tasks.

**Supplementary Information:**

The online version contains supplementary material available at 10.1007/s11571-024-10183-0.

## Introduction

The human brain is continuously exposed to a wide range of sensory stimuli originating from various modalities. The integration of these signals, particularly those from visual and auditory inputs, is essential for a variety of human behaviors (Ernst and Bulthoff 2004; Ferri et al. 2018; Cao et al. 2019; Hirst et al. 2020; Keil 2020; Senkowski and Engel 2024), including processes such as language processing (Conrey and Pisoni 2006; Francisco et al. 2017; Kaganovich 2017) and social perception (Brandwein et al. 2015; Noel et al. 2018). However, the neural mechanisms that underpin this audio-visual (A-V) integration remain insufficiently elucidated.

Several researchers have investigated the issue of temporal integration windows (TIW) in the context of bimodal (auditory and visual) input. It is well-established that temporal synchrony serves as a significant cue for auditory-visual (A-V) integration (Bauer et al. 2020; Zhou et al. 2020). When visual and auditory stimuli occur within a limited TIW, they are perceived as a unified event; conversely, stimuli that fall outside this interval are interpreted as distinct occurrences. Consequently, elucidating the neural underpinnings of the TIW is a crucial endeavor for understanding the mechanisms underlying A-V integration. Research findings within this field have demonstrated a degree of inconsistency and controversy. Oscillatory brain signals, particularly the phase and frequency of alpha rhythms (8–12 Hz), have been associated with the TIW (Cecere et al. 2015; Keil and Senkowski 2017; Cooke et al. 2019; Ikumi et al. 2019; Venskus and Hughes 2021; London et al. 2022; Ronconi et al. 2023). However, a recent study has challenged this viewpoint (Buergers and Noppeney 2022). The roles of beta (13–30 Hz) and gamma (> 30 Hz) rhythms have also been reported (Senkowski et al. 2007; Naue et al. 2011; Keil et al. 2014; Balz et al. 2016; Yuan et al. 2016; Kaiser et al. 2019; Theves et al. 2020; Michail et al. 2021, 2022; Jiang et al. 2024), along with a model addressing cross-frequency coupling (Lennert et al. 2021).

Another line of studies have investigated A-V integration by assessing the phase resetting of neural responses in the visual or auditory regions of brain. For instance, Kambe et al. (2015) conducted an experiment in which they recorded electroencephalogram (EEG) signals from human participants during a simultaneity judgment (SJ) task. In this task, participants were presented with an auditory stimulus (a beep) and a visual stimulus (a flash) that were separated by a short stimulus-onset asynchrony (SOA). Participants were instructed to determine whether they perceived the stimuli as occurring simultaneously. The findings indicated that when the initial auditory stimuli caused phase resetting of oscillatory signals in the visual cortex corresponding to the subsequent visual stimulus, participants were more likely to perceive the audiovisual stimuli as simultaneous. This phenomenon of cross-modal resetting, specifically from auditory to visual stimuli in this context, was mainly observed in the beta frequency band.

**Fig. 1:**
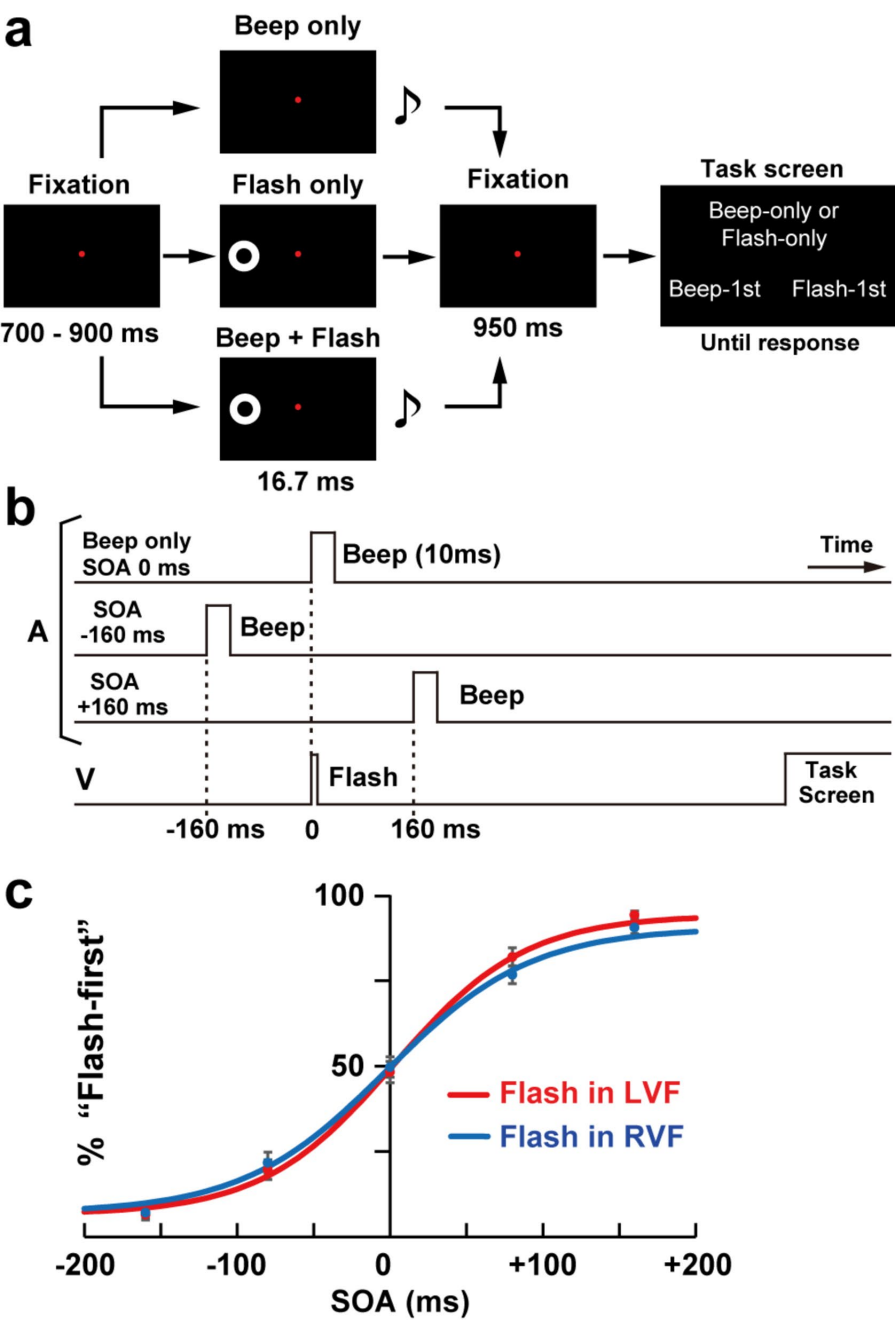
Stimuli and behavioral results. (**a**) Structures of trials. Every trial started with a fixation screen of 700–900 ms, followed by a beep (pure tone, frequency: 1800 Hz, duration: 10 ms), a flash (white ring, outer diameter: 5°, inner diameter: 2.5°, duration: 1 frame of a CRT monitor), or both. A task screen appeared after another fixation period of 950 ms. Participants pressed a button (“Up” key) to the beep-only and flash-only trials. When bimodal (beep + flash) inputs were given, they performed a temporal order judgment (TOJ) task, pressing “Left” key for the beep-first trials and “Right” key for the flash-first trials. A position of the flash was in left visual field (eccentricity: 5°) for three experimental sessions (flash-LVF sessions), while it was presented in right visual field for the other three (flash-RVF sessions). (**b**) Stimulus sequences. A stimulus onset asynchrony (SOA) between the beep and flash was − 160 ms, -80 ms, 0 ms, + 80 ms, or + 160 ms (negative: beep first). EEG data from − 1600 to + 1600 ms relative to a flash onset (at 0 ms) were analyzed. (**c**) Results of the TOJ task to bimodal inputs. Percentages of the “flash-first” response monotonically increased as a change in SOA. Points and error bars denote means and standard errors (SEs) of 28 participants, respectively. Curves with solid lines show results of the fitting by sigmoid functions

In the present study, we have expanded upon the research conducted by Kambe et al. (2015) by focusing on the following two points. First, our participants performed a temporal order judgment (TOJ) task, in contrast to the simultaneity judgment (SJ) task employed by Kambe et al. (2015). The TOJ task, which involved the simultaneous presentation of an auditory beep and a visual flash (see Fig. 1), provides an additional framework for exploring audiovisual (A-V) integration (Binder 2015; Zhou et al. 2020). If our research reveals a contribution of beta rhythm resetting within the TOJ task, it would imply that beta oscillations serve a more extensive, task-independent role in the auditory-visual (A-V) integration process. Secondly, while the flash in the study by Kambe et al. (2015) was presented within the central visual field, our study involved the presentation of the flash in either the left or right visual field (eccentricity: 5 deg in visual angle). This approach enabled us to manipulate the hemisphere (left or right) responsible for processing the visual information, potentially clarifying the lateralization of brain regions involved in A-V integration. Notably, we are particularly interested in the multisensory areas associated with A-V integration located in the parietal and posterior temporal cortices (Noesselt et al. 2007; Binder 2015; Lerousseau et al. 2022).

Consistent with previous studies (Kayser et al. 2008; Busch et al. 2009; Kambe et al. 2015), the phase resetting of EEG waveforms was quantified through the use of inter-trial coherence (ITC). ITC functions as a measure of phase coherence among oscillatory signals across various trials. A higher ITC value indicates that ongoing oscillations are influenced by sensory stimuli, which may be associated with an increase in neuronal firing rates (Lakatos et al. 2009). Participants were presented with either unimodal stimuli (beep or flash) or bimodal stimuli (beep + flash) while performing a temporal order judgment (TOJ) task associated with the bimodal stimuli. Drawing upon insights from animal research (Wallace et al. 2006; Kayser and Logothetis 2009; Meredith et al. 2020; Merrikhi et al. 2022), we hypothesized that human multisensory regions would demonstrate a higher ITC in response to bimodal stimuli compared to unimodal stimuli. Furthermore, if these regions possess the capability to detect the temporal alignment between visual and auditory stimuli through phase resetting, we would expect the ITC to increase as the stimulus onset asynchrony (SOA) approaches 0 ms. Lastly, we predicted that phase resetting would be more pronounced in the right hemisphere when the flash stimulus was presented in the left visual field (flash-LVF session, see **Materials and methods**), given that the visual processing of the flash primarily occurs in the contralateral right hemisphere. Conversely, when the flash was presented in the right visual field (flash-RVF session), phase resetting would be observed in the left hemisphere, where auditory and visual information converge.

## Materials and methods

### Participants

Thirty-one healthy subjects (age: 18–41 years, 14 females) participated in the present study. Data from three participants (2 males and 1 females) were excluded from the analysis due to issues with fitting behavioral data to a psychometric curve (*N* = 2) and the presence of noise in EEG waveforms (*N* = 1). Consequently, the final dataset comprised 28 participants, a number that aligns with recent EEG research examining audiovisual interaction (Ikumi et al. 2019; Noguchi 2022; Ronconi et al. 2023; Sciortino and Kayser 2023). Each participant exhibited normal auditory and visual capabilities. The objectives of the study were explicitly communicated to all participants, and informed consent was subsequently acquired. All experimental procedures adhered to the ethical standards and regulations established by the ethics committee at Kobe University, Japan.

### Stimuli and task

All visual and auditory stimuli were generated using Matlab Psychophysics Toolbox (Brainard 1997; Pelli 1997) and were presented on a CRT monitor (refresh rate: 60 Hz) or by two speakers (intensity: 77 dB SPL), with one each on either side of the CRT monitor. The visual stimulus consisted of a white ring, with an outer diameter of 5° and an inner diameter of 2.5°, presented for a duration of 16.67 ms (1 frame of the CRT monitor). The auditory stimulus was a pure tone beep (frequency = 1800 Hz, duration = 10 ms). These parameters of visual and auditory stimuli were based on prior research (Fujisaki et al. 2004; Kambe et al. 2015). Participants were instructed to maintain their gaze on a red fixation point (0.18 deg × 0.18 deg) located at the center of the screen throughout the duration of the experiment. As shown in Fig. 1a, each trial started with a fixation screen lasting between 700 and 900 ms, which was subsequently followed by either a beep, a flash, or a combination of beep-flash stimuli. For bimodal inputs, a SOA between the beep and flash was randomly assigned to one of five intervals: -160 ms, -80 ms, 0 ms, + 80 ms, or + 160 ms (negative: beep first, Fig. 1b). Following an additional fixation period of 950 ms, a task screen was presented. Participants were instructed to discriminate unimodal from bimodal inputs and then perform the TOJ task to the bimodal inputs. Specifically, they pressed an up (**↑)** key for the unimodal (beep-only and flash-only) trials. For bimodal inputs (SOAs from − 160 to + 160 ms), on the other hand, they pressed a left (←) key when an onset of the beep was perceived to precede that of the flash (“beep-first” responses) and pressed a right (→) key to indicate the reverse (“flash-first” responses).

Each experimental session contained 126 trials in which the seven types of trials (flash-only, beep-only, SOA − 160 ms, -80 ms, 0 ms, + 80 ms, and + 160 ms. 18 trials for each) were randomly intermixed. Participants performed six sessions in total. The flash stimulus was presented in the left visual field (eccentricity: 5°) during three of the experimental sessions (flash-LVF sessions), while it was presented in the right visual field during the remaining three sessions (flash-RVF sessions). The presentation delay between the two points on the cathode ray tube (CRT) monitor was verified to be less than 1 ms using a photodiode and an oscilloscope. Consequently, each of the 14 conditions (comprising the seven trial types across both flash-LVF and flash-RVF presentations) consisted of 54 trials (18 trials multiplied by 3 sessions). The sequence of flash-LVF and flash-RVF sessions was counterbalanced across participants.

### Analysis of behavioral data

The accuracy of the flash-only and beep-only conditions was determined by calculating the ratio of the number of up key presses during the presentation of flashes or beeps to the total number of trials conducted in the flash-only or beep-only conditions. For trials with bimodal inputs, changes in percentages of a “flash-first” response (%“Flash-1st”, Fig. 1c) along with SOA (-160 to + 160 ms) were fitted by a sigmoid function (Noguchi et al. 2011).

$$F(x) = Min + (Max - Min)/[1+e^{-a(x-b)}]$$

Here *x* was the SOA, *a* and *b* were free parameters obtained through the Nelder–Mead method. The Max and Min indicated the maximum and minimum %“Flash-1st” across five levels of SOA.

We assessed two measures of the fitted psychometric function; the point of subjective simultaneity (PSS) and the just-noticeable difference (JND). The PSS represents the SOA at which the fitted function reaches a 50%-threshold, indicating a point at which stimuli were perceived to occur simultaneously. The JND represents the minimal detectable asynchrony between the auditory beep and visual flash, as determined by the methodology outlined by Paire et al. (2023).

$$JND = (SOA_{75} - SOA_{25})/2$$

where SOA_75_ and SOA_25_ correspond to SOAs at which %“Flash-1st” was equal to 75 and 25, respectively. A larger JND indicates a shallower slope of the psychometric function and thus indexes a lower sensitivity to temporal asynchrony.

### EEG measurements

**Fig. 2:**
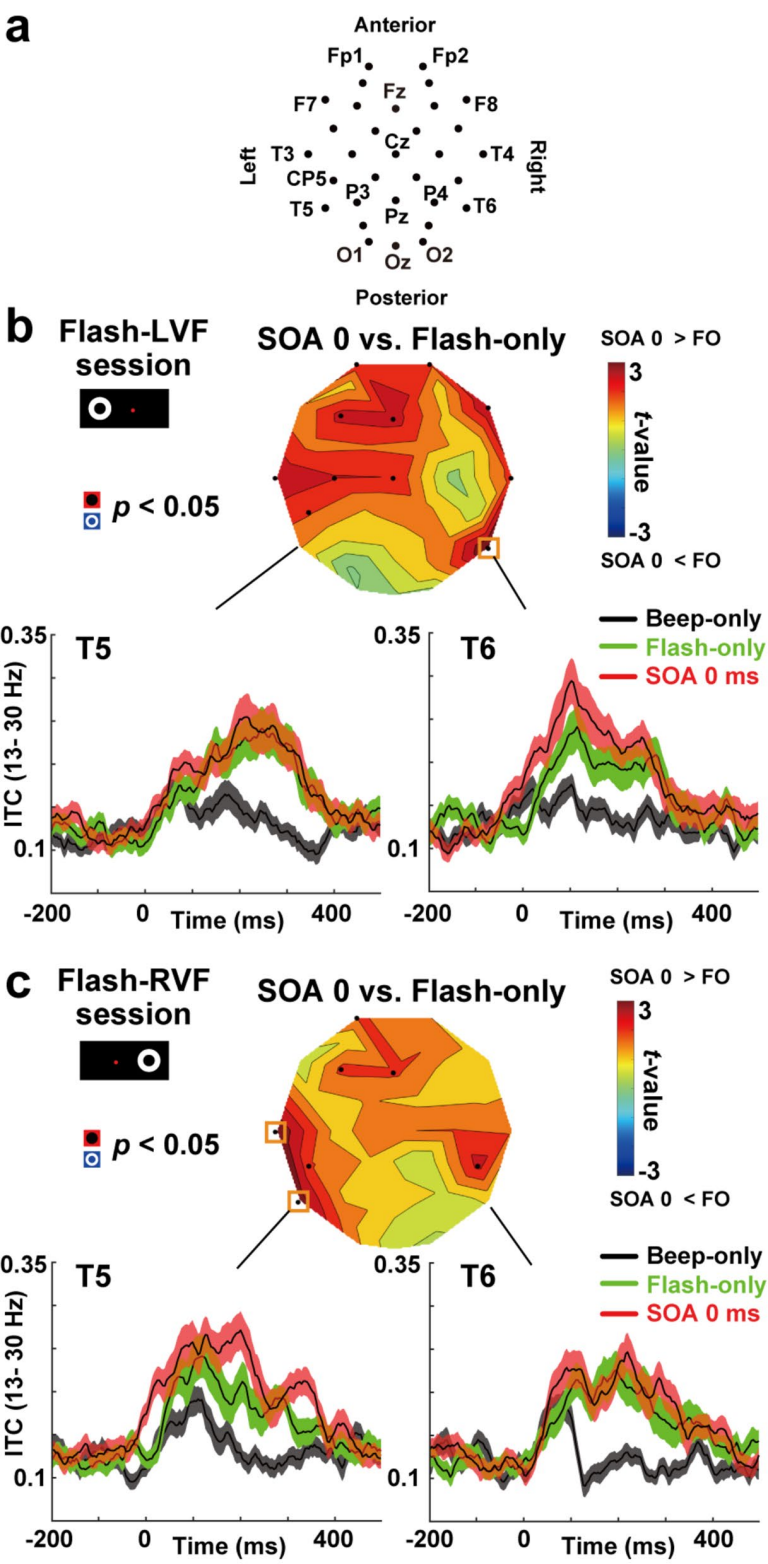
Inter-trial coherence (ITC) in beta band (13–30 Hz). (**a**) Two-dimensional layout of 32 sensors of EEG. (**b**) Upper panel: Statistical *t*-maps of mean ITC (0–100 ms) between SOA-0ms vs. flash-only (FO) trials in flash-LVF sessions, depicted over a layout of the 32 EEG sensors (upward: anterior). Black dots indicate sensors showing higher ITC to bimodal (SOA-0ms) than flash-only trials (*p* < 0.05, uncorrected). Orange rectangles denote a significant difference after a correction of multiple comparisons (*p* < 0.05, FDR corrected). Lower panels: Changes in beta ITC at T5 (left) and T6 (right) from − 200 to 500 ms. Background shadings denote SEs across participants. ITCs in SOA-0ms trials (red) were larger than those in flash-only trials (green) at T6, indicating that a beep concurrently given with a flash induced phase resetting of beta rhythm in the contra-flash (right) hemisphere. No such A-V integration was seen at T5 in the ipsi-flash (left) hemisphere. (**c**) Same as panel **b**, but the data in flash-RVF session are shown

EEG signals were recorded from 32 locations on the scalp (FP1, FP2, AF3, AF4, F7, F3, Fz, F4, F8, FC5, FC1, FC2, FC6, T3, C3, Cz, C4, T4, CP5, CP1, CP2, CP6, T5, P3, Pz, P4, T6, PO3, PO4, O1, Oz, and O2, see Fig. 2a). Measurements were carried out using an ActiveTwo EEG system (Biosemi, Amsterdam, Netherlands) with a sampling frequency of 2,048 Hz and an analog low-pass filter set at 417 Hz. Data processing was performed utilizing the Brainstorm toolbox for Matlab (Tadel et al. 2011), wherein all data were referenced to the average potential across the 32 electrodes. Artifacts and noise were corrected by notch (60, 120, and 180 Hz) and band-pass (0.5–200 Hz) filters. Subsequently, the EEG waveforms were segmented into each trial (epoch range: −1600 to 1600 ms relative to a flash onset at 0 ms). These trials were then classified into the 14 conditions, produced by the seven types of trials (flash-only, beep-only, SOA ± 160 ms, ± 80 ms, and 0 ms) × two types of sessions (flash-LVF and flash-RVF).

### Analysis of EEG data

The present study primarily focused on the analysis of inter-trial coherence (ITC). Initially, the raw electroencephalogram (EEG) waveforms were decomposed into six distinct frequency bands: delta (2–4 Hz), theta (5–7 Hz), alpha (8–12 Hz), beta (13–30 Hz), gamma (31–58 Hz), and high-gamma (62–100 Hz). For the beta band, a band-pass filter with a range of 13–30 Hz (zero-phase, Butterworth) was applied to the raw EEG data. Additionally, signals within the range of 58–62 Hz were excluded from the analysis to mitigate the influence of power-line noise at 60 Hz. The filtered waveforms were then transformed using the Hilbert transformation to derive the instantaneous phase changes over time. The ITC for each frequency band was subsequently calculated using the following equation,

$$\:{ITC}_{\left(t,f\right)}=\frac{1}{N}|\sum\:_{k=1}^{N}{e}^{i{\phi\:}_{k\left(t,f\right)}}|$$

where “*N*” represents the number of trials for each condition (54) and “*φk*(*t*,*f*)” denotes an instantaneous phase of frequency band “*f*” (from delta to high-gamma) at a time “*t*” (− 1600 to 1600 ms) of trial “*k*” (from 1 to 54). The ITC ranges from 0 to 1, with a value of 0 signifying a lack of phase synchronization across trials, and a value of 1 indicating complete synchronization. A baseline correction for the ITC was not implemented due to the extensive range of SOAs (-160 to + 160 ms), which complicated the establishment of a uniform baseline period applicable to all conditions. Nevertheless, we verified that our primary findings remained consistent even when a baseline correction was applied using data from − 200 to 0 ms.

We initially examined the differences in ITC between unimodal and bimodal conditions. The ITC in SOA-0ms was compared with flash-only and beep-only trials (Fig. 2). If the responses of multisensory regions were accurately represented in the ITC, then the simultaneous presentation of flash and beep stimuli in the SOA-0ms trials would result in a resetting of the current phase, thereby producing a higher ITC compared to the unimodal flash and beep trials. This increase in ITC would predominantly manifest in the right hemisphere in the flash-LVF session (Fig. 2b), as the visual information from the flash would be processed in the contralateral right hemisphere. Conversely, A-V integrationin the flash-RVF session (Fig. 2c) would be observed in the left hemisphere, where auditory information converged with visual information.

### Statistical procedures

To test the aforementioned prediction, we calculated the mean ITC at 0–100 ms and compared those between SOA-0ms vs. flash-only trials. This time window (0–100 ms) was based on a prior research conducted by Kambe et al. (2015). If A-V integration occurred in the ITC, a beep presented simultaneously with a flash would elicit a greater ITC response in the hemisphere opposite the flash, compared to when only the flash was presented. This phenomenon would be indexed by reddish color on the *t*-maps (Fig. 2), which illustrate the mean ITC for the SOA-0ms trials in contrast to the flash-only trials. To address the issue of multiple comparisons arising from the repeated paired t-tests conducted across 32 sensor positions, we employed the false discovery rate (FDR) methodology (Noguchi 2024), which involved adjusting the significance threshold using the Benjamini–Hochberg correction (Benjamini and Hochberg 1995). The sensors that exhibited statistically significant differences following this correction are highlighted with orange rectangles.

In a secondary analysis, we conducted a comparison of ITC in the flash-LVF session with that in the flash-RVF sessions. As illustrated in Fig. 4, we analyzed the mean ITCs (0–100 ms) across beep-only (top panel), flash-only (middle panel), and SOA-0ms trials (bottom panel) between the flash-LVF and flash-RVF sessions. If phase resetting of the beta rhythm takes place within the multisensory regions, this analysis would demonstrate a lateralization of activity in these areas during the SOA-0 ms trials. In particular, synchronous audiovisual stimuli presented in the LVF session would be expected to produce elevated ITC in the right hemisphere (indicated by reddish hues). Conversely, stimuli presented in the RVF session are expected to lead to increased ITC in the left hemisphere (indicated by reddish hues).

## Results

### Behavioral data

Accuracy in beep-only trials (mean ± SE across subjects) was 99.74 ± 0.12% in flash-LVF and 99.54 ± 0.18% in flash-RVF sessions. Accuracy in flash-only trials was 99.01 ± 0.51% in flash-LVF and 99.47 ± 0.21% in flash-RVF sessions.

Figure 1c shows the averaged psychometric curve for %“Flash-1st” fitted by a sigmoid function. In both flash-LVF and flash-RVF sessions, the %“Flash-1st” monotonically increased as a change in SOA. The PSSs averaged across participants were 0.16 ± 6.79 ms in flash-LVF and 0.62 ± 6.89 ms in flash-RVF sessions. Mean JNDs were 56.11 ± 6.94 ms in flash-LVF and 61.73 ± 5.93 ms in flash-RVF sessions. No significant difference was observed between flash-LVF and -RVF sessions either in PSS (*t*(27) = 0.09, *p* = 0.93, Cohen’s *d* = 0.01) or JND (*t*(27) = 0.74, *p* = 0.46, *d* = 0.16).

### Comparisons of ITC between SOA-0ms and flash only trials

Based on previous studies (Kayser and Logothetis 2009; Kambe et al. 2015), we mainly focused on the ITC in beta band (13–30 Hz). Results in other frequency bands (delta, theta, alpha, gamma, and high-gamma) were provided in Figs. S1 in Supplementary materials.

Figure 2b shows beta ITC (13–30 Hz) in the flash-LVF sessions. The ITC in SOA-0ms bimodal trials (red line) was larger than those in unimodal flash-only (green) and beep-only (black) trials over the right hemisphere under T6 (lower right panels). No such difference was observed in the ipsi-flash left hemisphere under T5 (lower left panels). In the flash-RVF sessions (Fig. 2c), reversed laterality was observed. The ITC in SOA-0ms trials (red) was larger than those in flash-only and beep-only trials over the left hemisphere under T5 (left panels).

We statistically examined this point by drawing *t*-maps of mean ITCs (0–100 ms) between SOA-0ms vs. flash-only trials (upper panels in Fig. 2b and c). Sensors with a significant difference after a correction of multiple comparisons are indicated by orange rectangles. In addition to auditory responses over the front-midline regions (Carral et al. 2005; Stefanics et al. 2009; Tong et al. 2009; Peter et al. 2010; Kambe et al. 2015; Noguchi et al. 2015), SOA-0ms trials elicited an increase in beta ITC over the temporal cortex. When a flash was in LVF (Fig. 2b), we found higher ITC at a right temporal electrode (T6: *t*(27) = 3.68, *p* = 0.001, *d* = 0.75). When the flash was in RVF (Fig. 2c), higher ITC was seen at left temporal electrodes (T3: *t*(27) = 3.51, *p* = 0.002, *d* = 0.90, T5: *t*(27) = 3.30, *p* = 0.003, *d* = 0.55).

A recent study reported changes in beta rhythm related to A-V integration at two separate periods after a stimulus onset; an early period at 0–90 ms and a late period at 250–380 ms (Michail et al. 2021). While the early period is substantially overlapped with our analysis window (0–100 ms), beta ITC at the late period remains to be analyzed. In Fig. S2 in Supplementary materials, we calculated mean ITC at 250–380 ms, finding larger ITC in SOA-0ms than flash-only trials. These data showed a persistent change of beta ITC to bimodal (A + V) inputs, continuously seen both in the early and late periods after task stimuli.

### Comparisons of ITC over five SOAs

**Fig. 3:**
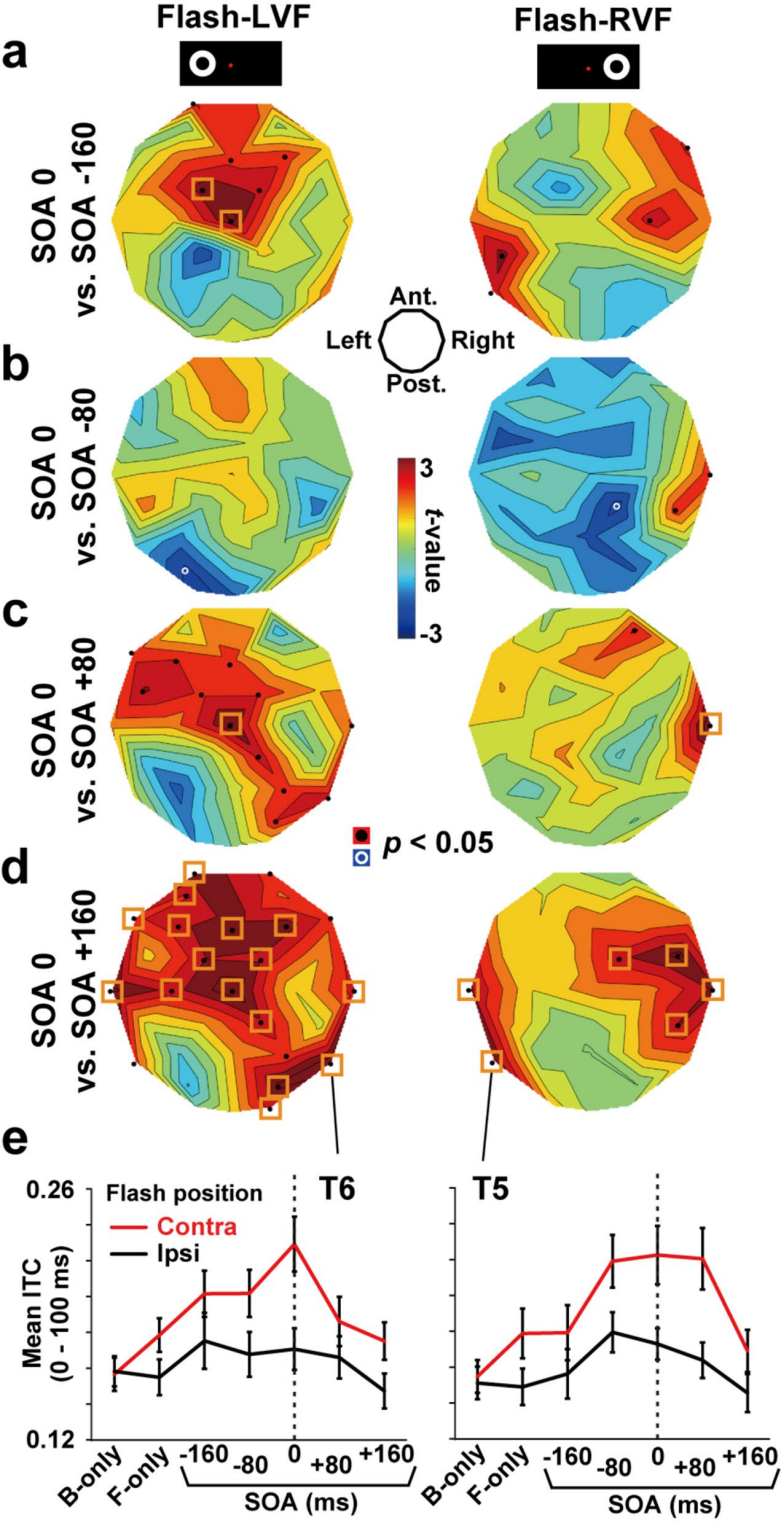
Changes in beta ITC over five SOAs from − 160 to + 160 ms. The *t*-maps of mean ITC (0–100 ms) between SOA 0 vs. -160 ms (**a**), between SOA 0 vs. -80 ms (**b**), between SOA 0 vs. +80 ms (**c**), and between SOA 0 vs. +160 ms (**d**) are shown. In the flash-LVF session (left panels), *t*-values at T6 were positive in all maps, indicating that the SOA-0ms trials induced the highest ITC over the five SOAs. Similar results are seen at T5 in the flash-RVF session (right panels). (**e**) Mean ITCs at T6 and T5 in all 14 conditions. Error bars denote SEs across participants. The highest ITC was seen in SOA-0ms trials (dotted lines) when an auditory input converged with a visual input in the contra-flash hemisphere

We then tracked changes in beta ITC across five SOAs from − 160 to 160 ms. The *t*-maps comparing SOA 0 ms with SOAs − 160 ms, -80 ms, + 80 ms, and + 160 ms are shown in Fig. 3a, b, c, and 3d, respectively. In the flash-LVF session (left panels), *t*-values at T6 (over the right temporal cortex) were positive in all maps, indicating that the SOA-0ms trials induced the highest ITC over the five SOAs. Similar results were seen at T5 (left temporal cortex) in the flash-RVF session (right panels).

Figure 3e summarizes mean beta ITC (13–30 Hz, 0–100 ms) at T6 and T5 in all 14 conditions. Mean ITCs in the contra-flash conditions (red) were higher than those in the ipsi-flash conditions (black). The highest ITC was observed in SOA-0ms trials (dotted line), reflecting strong phase resetting to synchronous A-V inputs.

### Comparisons of ITC between flash-LVF and flash-RVF sessions

**Fig. 4:**
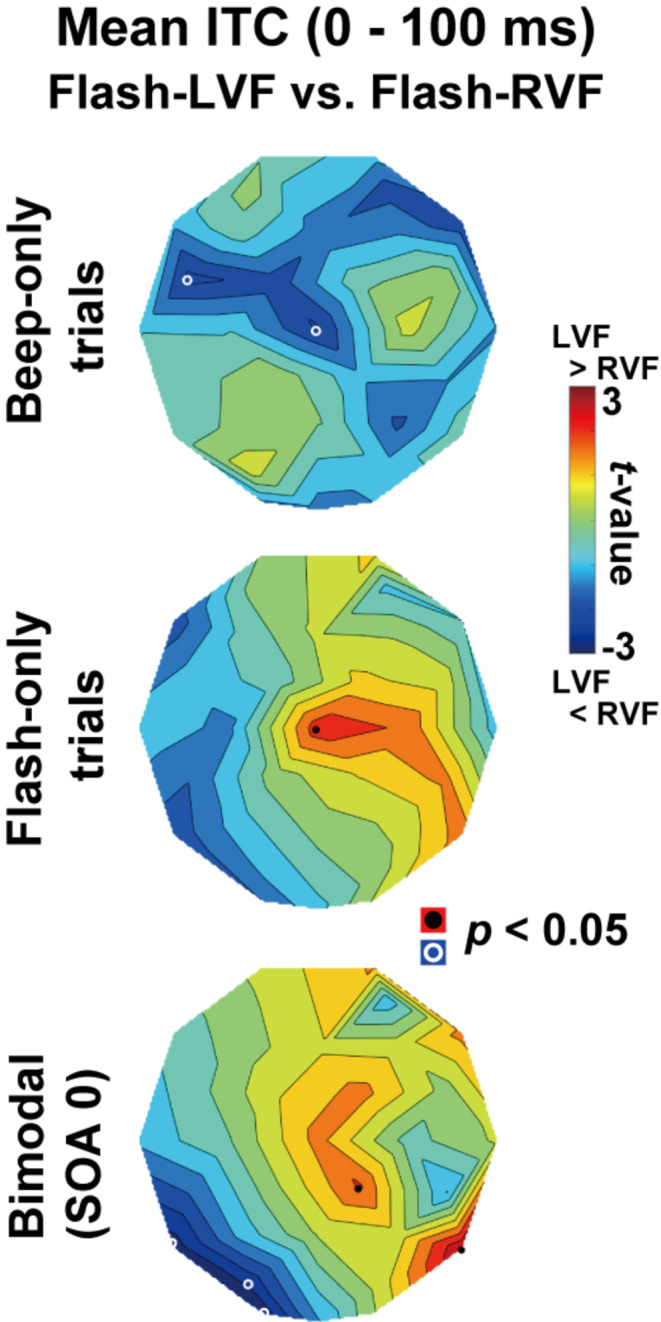
Comparisons of beta ITC (0–100 ms) between Flash-LVF vs. Flash-RVF sessions. Top panel: Beep-only trials. Middle panel: Flash-only trials. Bottom panel: Bimodal (SOA-0ms) trials. Black dots and white circles indicate differences (Flash-LVF minus Flash-RVF) of *p* < 0.05 (uncorrected). In SOA-0ms trials, beta ITC showed an increase at temporal sensors in a contra-flash hemisphere, although these differences did not reach significance after the correction of multiple comparisons (see **Results** for details)

Finally, we compared beta ITC between flash-LVF and -RVF sessions to see the laterality of the multisensory areas (Fig. 4). The *t*-map of SOA-0ms trials (bottom panel) showed differences in ITC (flash-LVF > RVF, red) over the right posterior region and differences in an opposite direction (flash-LVF < RVF, blue) over the left posterior regions. However, the difference at T5 (*t*(27) = -2.82, *p* = 0.009, *d* = -0.72) or T6 (*t*(27) = 3.05, *p* = 0.005, *d* = 0.82) did not reach significance after the correction of multiple comparisons.

**Fig. 5:**
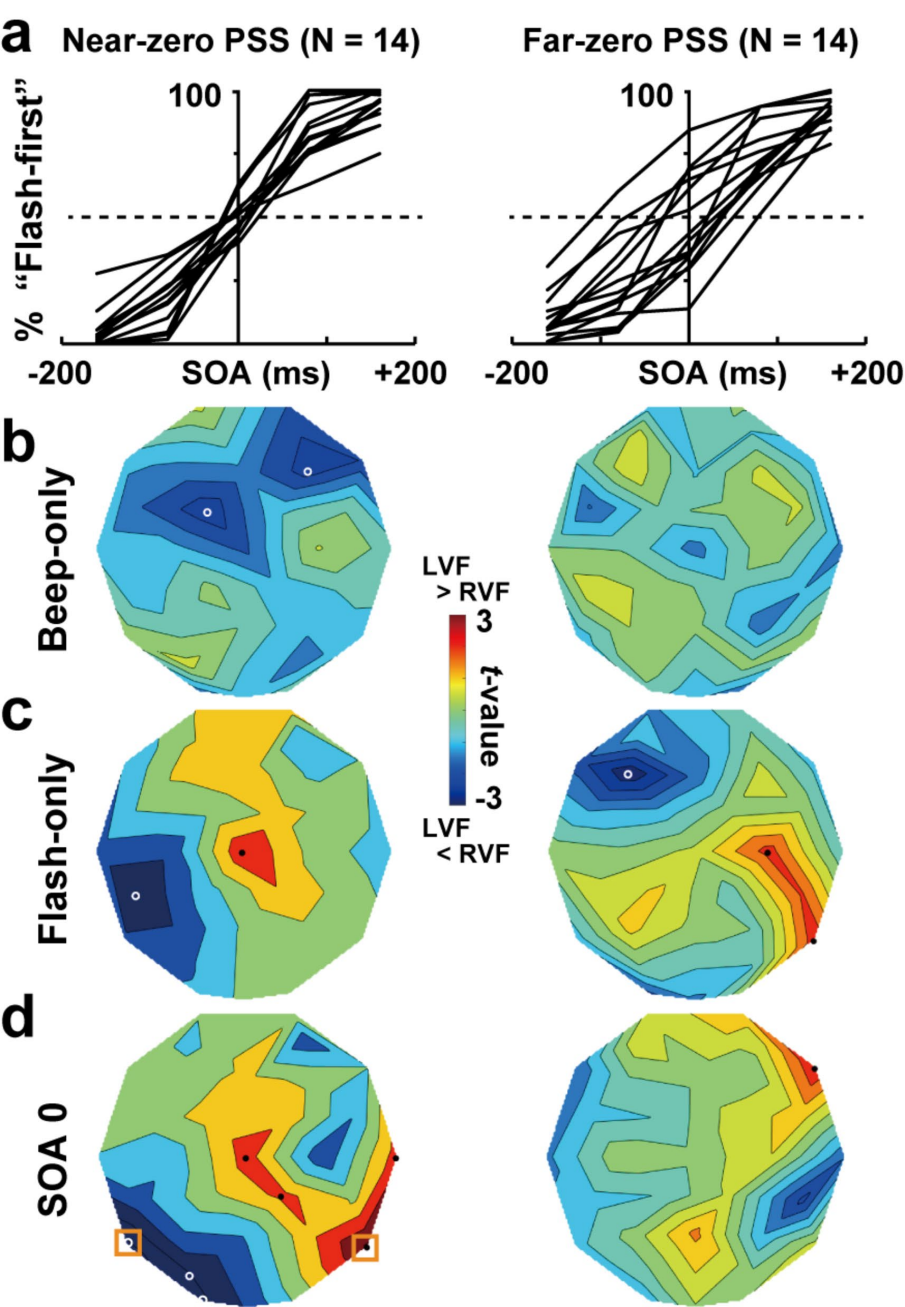
Comparisons of near-zero PSS (point of subjective simultaneity) and far-zero PSS participants. (**a**) Individual behavioral data (psychometric curves). The 28 participants were classified into two groups based on a median split of their absolute PSSs. (**b**) *t*-maps of beta ITC (0–100 ms) in beep-only trials (Flash-LVF session minus Flash-RVF session). Left and right panels showed the maps for near-zero PSS and far-zero PSS participants, respectively. (**c**) *t*-maps of beta ITC in flash-only trials. (**d**) *t*-maps of beta ITC in SOA-0ms trials. The laterality was more distinct in the near-zero PSS group (left) than far-zero PSS group (right), suggesting that clear ITC change in the contra-flash hemisphere was associated with accurate TOJ

One possibility for this lack of significance might be a large inter-individual variability in behavioral data of the TOJ task (Grabot et al. 2017). We thus classified 28 participants into two groups depending on their absolute PSSs (Fig. 5a). The absolute PSS of each participant was obtained from his/her psychometric curve in which all (flash-LVF + flash-RVF) trials were combined. Fourteen participants in the near-zero PSS group were characterized by PSSs around 0 ms (absolute PSS: 8.26 ± 1.20 ms, upper left panel). Subjective simultaneity of those participants was tuned to objective simultaneity (0 ms), suggesting that they were highly sensitive to simultaneous A-V inputs. Consistent with behavioral data, a comparison of beta ITC (flash-LVF vs. RVF) in SOA-0ms trials (Fig. 5d, left panel) revealed a clear laterality. When a beep was presented simultaneously with a flash in LVF, ITC over the right temporal cortex showed a prominent increase (shown in red, T6: *t*(13) = 4.19, *p* = 0.001, *d* = 1.24). The beep synchronized with the flash in RVF evoked ITC over the left temporal cortex (blue, T5: *t*(13) = -4.36, *p* = 0.0008, *d* = -1.28). No clear change was observed in ITC of far-zeros PSS participants (absolute PSS: 39.88 ± 6.25 ms, right panels). Note that, although participants in those two groups were highly different in their absolute PSSs (*t*(26) = 4.97, *p* < 0.001, *d* = 1.88), no difference was observed in their JNDs (near-zero PSS group: 57.74 ± 9.04 ms, far-zero PSS group: 65.37 ± 7.53 ms, *t*(26) = 0.65, *p* = 0.52, *d* = 0.25).

We also performed the same (median-split) analysis based on JND data, dividing 28 participants into small JND (*N* = 14) and large JND (*N* = 14) groups. As shown in Fig. S3 in Supplementary materials, the laterality in SOA-0ms trials tended to be clearer in the small JND than large JND groups, although no electrode showed a significant difference (Flash-LVF vs. Flash-RVF) after the correction of multiple comparisons.

## Discussion

In the present study, we measured human EEG signals during a temporal-order judgment task involving auditory and visual stimuli. In contrast to prior research that focused on event-related potentials (ERPs) or steady-state responses (Mishra et al. 2007; Covic et al. 2017; Lerousseau et al. 2022; Gori et al. 2023; Sciortino and Kayser 2023), this study employed ITC to identify neural rhythms that exhibit phase resetting in response to multisensory inputs. Compared to unimodal conditions (beep-only and flash-only trials), bimodal inputs in SOA-0ms trials resulted in a increase in beta ITC over the posterior temporal region contralateral to the location of a flash (Fig. 2). Additionally, participants who demonstrated a pronounced laterality in the ITC increase (flash-LVF vs. flash-RVF) exhibited a PSS close to 0 ms (Fig. 5). These findings suggest that the phase resetting associated with bimodal inputs facilitates an accurate temporal-order judgment, thereby allowing for a precise alignment of subjective simultaneity with objective simultaneity between auditory and visual stimuli.

The findings of this study align with prior research that has identified a significant role for beta rhythm in A-V integration (Kayser and Logothetis 2009; Naue et al. 2011; Kambe et al. 2015; Michail et al. 2021; Noguchi 2022). A notable hallmark of our research was the implementation of two varieties of temporal order judgment (TOJ) trials, during which a flash was displayed in either the left or right visual fields. This methodological approach facilitated the manipulation of the hemisphere in which auditory inputs were integrated with visual stimuli. This procedure revealed that phase resetting of the beta rhythm occurred selectively in the hemisphere contralateral to the flash presentation (Fig. 2), with a pronounced effect observed over the posterior temporal cortex. These results were in line with previous studies (Noesselt et al. 2007; Lerousseau et al. 2022)that have documented A-V multisensory responses within the human temporal cortex. The observed increase in ITC might be indicative of phase resetting of membrane potentials in these regions, which is likely a consequence of heightened neuronal firing rates in response to bimodal inputs (Lakatos et al. 2009).

Numerous studies have indicated the significance of phase resetting in the cross-modal interaction between visual and auditory modalities (Bauer et al. 2020; Brang et al. 2022). However, the majority of these investigations have focused on “cross-modal transmission” rather than “cross-modal integration,” which is the emphasis of the current study. For instance, Plass et al. (2019) conducted electrocorticographic (ECoG) recordings and observed an increase in ITC within the visually-responsive cortex in response to auditory stimuli (Plass et al. 2019). While these findings demonstrated a cross-modal transmission of unimodal inputs from auditory to visual systems, it remained ambiguous whether phase resetting was also associated with the integrative (conjunctive) processing of bimodal inputs (A + V). In this context, our data revealed alterations in ITC across the human posterior cortex, which functions as a temporal integrator (coincidence detector) for visual and auditory inputs.

On the other hand, The current study is subject to several limitations, which are outlined below. Firstly, the spatial resolution of scalp EEG was relatively low. While we observed an increase in ITC during bimodal trials compared to unimodal trials at the temporal electrodes (T5 and T6), it is essential to accurately identify the anatomical source locations of these responses using methodologies that offer superior spatial resolution, such as electrocorticography (ECoG). Secondly, the findings of this study do not preclude the possibility that oscillatory signals beyond the beta rhythm may play a role in A-V integration. For example, we observed an marginal increase (*p* < 0.05 uncorrected) in alpha ITC for bimodal inputs compared to unimodal inputs over the temporal cortex (Fig. S1), although this increase did not reach statistical significance after the correction of multiple comparisons. We hypothesize that this marginal significance in alpha ITC may be linked to the ongoing debate regarding the alpha rhythm and TIW in a previous literature (see Introduction). Although changes in beta ITC was most clearly seen in the present data, oscillatory signals in other frequency bands warrant further investigation in future research.

## Electronic Supplementary Material

Below is the link to the electronic supplementary material.

Supplementary Material 1

## Data Availability

The data presented in this study are available on request from the corresponding author.
