# Supplementary material for: The role of beta band phase resetting in audio-visual temporal order judgment
Source: Cogn Neurodyn. 2025 Jan 15;19(1):28. doi: 10.1007/s11571-024-10183-0 (PMC11735826; doi:10.1007/s11571-024-10183-0)
Supplement: Supplementary file 1 — Supplementary Material 1 [file 11571_2024_10183_MOESM1_ESM.pdf]

Supplementary materials (Figs. S1-S3)

The role of beta band phase resetting in audio-visual temporal order judgment

Yueying Li and Yasuki Noguchi

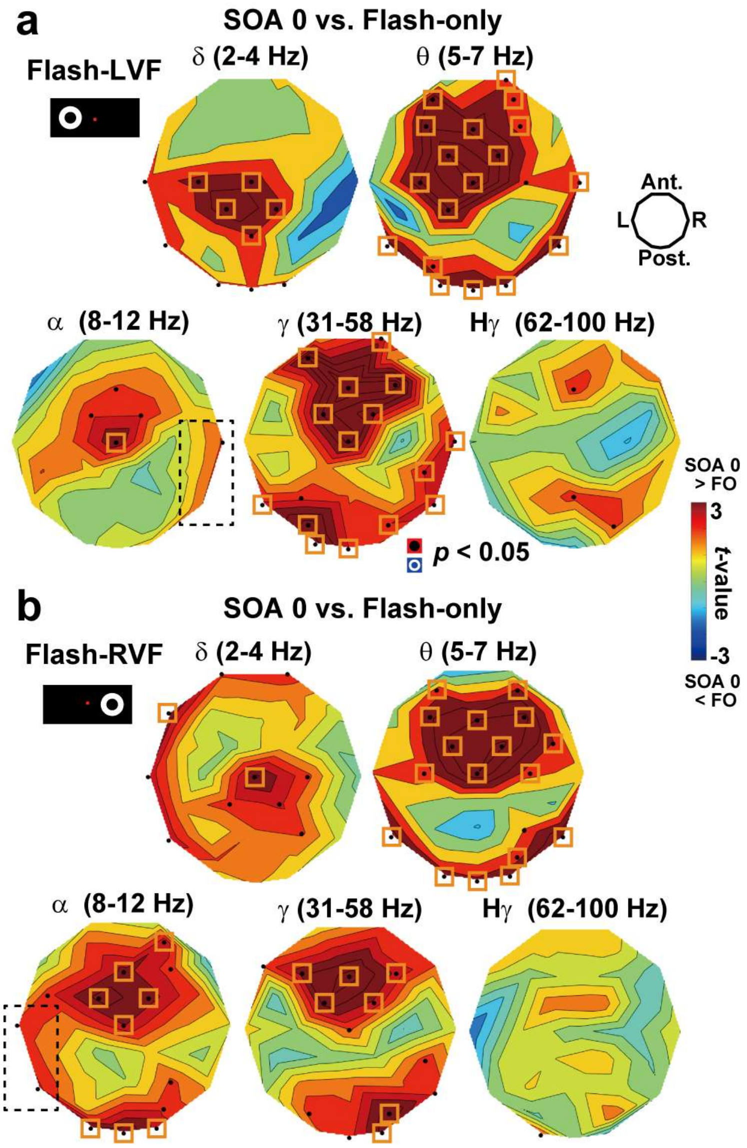

Fig. S1 Inter-trial coherence (ITC) in delta (2 – 4 Hz), theta (5 – 7 Hz), alpha (8 – 12 Hz), gamma (31 – 58 Hz), and high-gamma (62 – 100 Hz) bands. (**a**) Statistical *t*-maps of mean ITC (0 – 100 ms) between SOA-0ms vs. flash-only (FO) trials in flash-LVF sessions. Black dots indicate sensors showing higher ITC to bimodal (SOA-0ms) than FO trials ($p < 0.05$, uncorrected). Orange rectangles denote a significant difference after a correction of multiple comparisons ($p < 0.05$, FDR corrected). (**b**) Same as panel **a**, but the data in flash-RVF session are shown. A beep in SOA-0ms trials elicited increases in alpha ITC (SOA-0ms > FO) over the temporal cortex of the contra-flash hemisphere (dotted rectangles), although those differences did not reach significance after the FDR correction. Higher alpha ITC (SOA-0ms > flash-only) were observed in flash-LVF session at T4 ($t(27) = 2.10$, $p = 0.045$, $d = 0.48$) and T6 ($t(27) = 2.04$, $p = 0.052$, $d = 0.24$) and in flash-RVF sessions at T3 ($t(27) = 2.27$, $p = 0.03$, $d = 0.51$) and T5 ($t(27) = 2.60$, $p = 0.02$, $d = 0.35$).

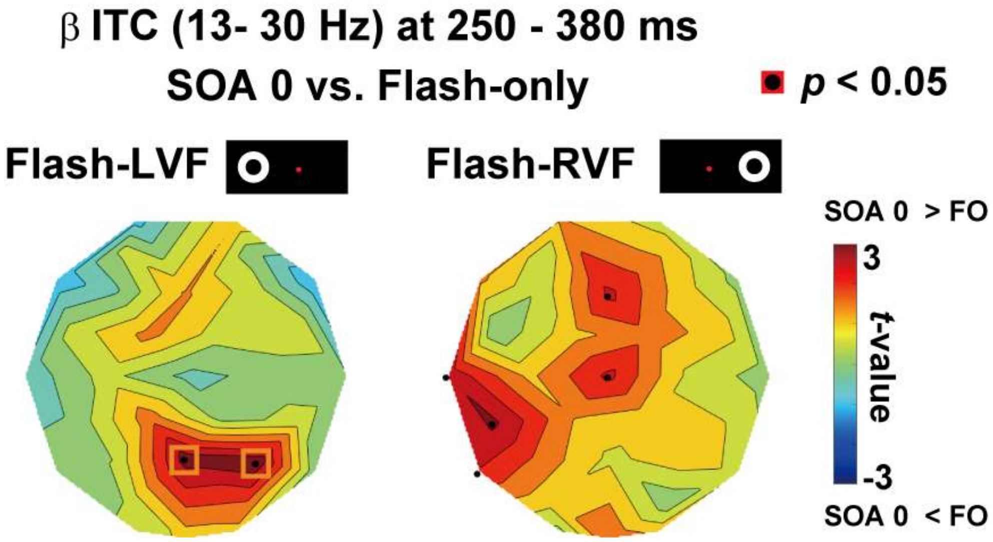

Fig. S2 Beta ITC in a late period. Mean ITC at 250 - 380 ms were compared between SOA-0ms vs. flash-only trials. Consistent with **Fig. 2** (0 – 100 ms), beta ITC in SOA-0ms trials was larger than that in flash-only trials in the contra-flash hemisphere.

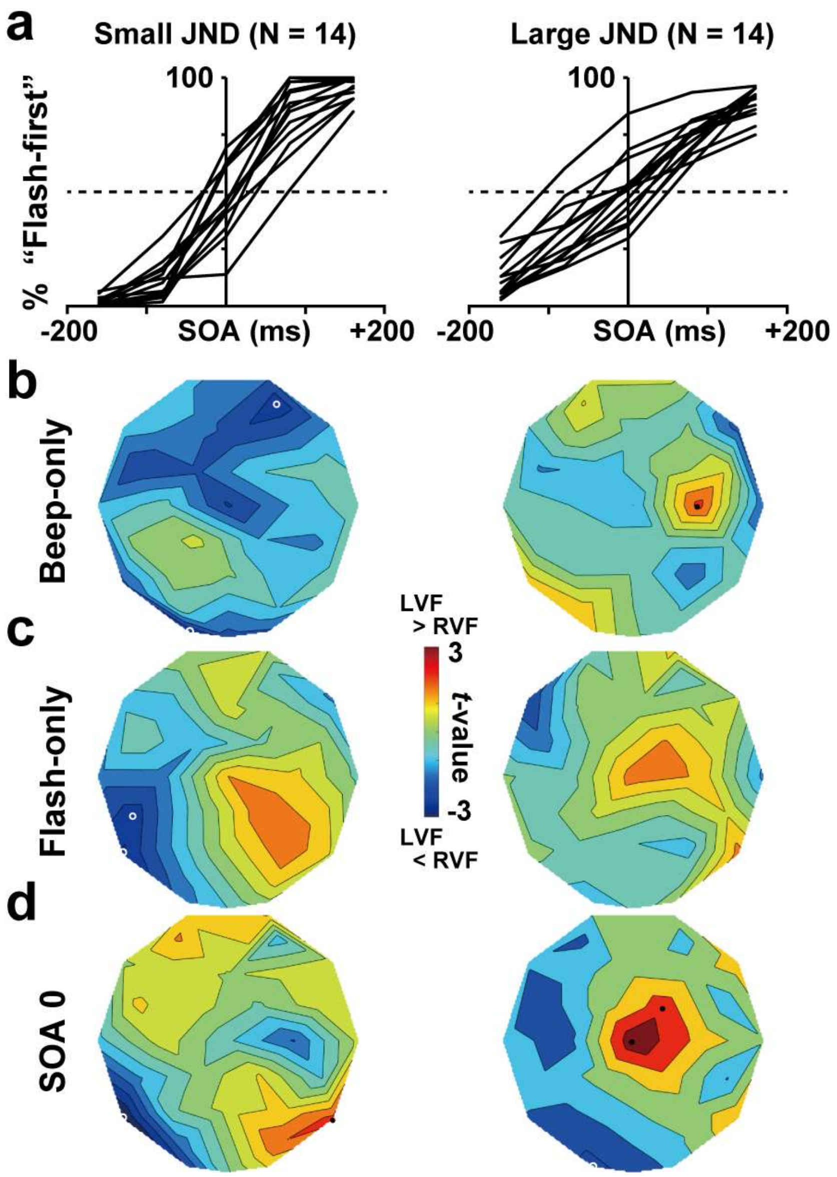

Fig. S3 Comparisons of small JND (just-noticeable difference) and large JND participants. (**a**) Individual behavioral data (psychometric curves). The 28 participants were classified into two groups based on a median split of their JNDs. Although participants in those two groups were highly different in their JNDs (small JND group: 40.77 ± 3.52 ms , large JND group: 82.34 ± 7.86 ms, $t(26) = 4.83$, $p < 0.001$, $d = 1.82$), no difference was observed in their absolute PSSs (small JND group: 20.03 ± 5.15 ms , large JND group: 28.11 ± 7.07 ms, $t(26) = 0.92$, $p = 0.36$, $d = 0.35$) (**b**) *t*-maps of beta ITC (0 – 100 ms) in beep-only trials (Flash-LVF session minus Flash-RVF session). Left and right panels showed the maps for small JND and large JND participants, respectively. (**c**) *t*-maps of beta ITC in flash-only trials. (**d**) *t*-maps of beta ITC in SOA-0ms trials. The laterality tended to be clearer in the small than large JND groups, although no electrode showed a significant difference after the correction of multiple comparisons.
